# Comparison of the Conceptual Map and Traditional Lecture Methods on Students’ Learning Based on the VARK Learning Style Model: A Randomized Controlled Trial

**DOI:** 10.1177/2377960820940550

**Published:** 2020-07-08

**Authors:** Sara Amaniyan, Vahideh Pouyesh, Yousef Bashiri, Sherrill Snelgrove, Mojtaba Vaismoradi

**Affiliations:** 1 Student Research Center, Semnan University of Medical Sciences, Iran; 2 Faculty of Nursing and Midwifery, Iranshahr University of Medical Sciences, Iran; 3 Department of Biostatistics, School of Allied Medical Sciences, Shahid Beheshti University of Medical Sciences, Tehran, Iran; 4 Department of Public Health, Policy and Social Sciences, Swansea University, UK; 5 Faculty of Nursing and Health Sciences, Nord University, Bodø, Norway

**Keywords:** conceptual map, learning styles, nursing student, teaching methods, VARK model

## Abstract

Developing skills and knowledge in nursing education remains a considerable challenge. Nurse instructors need to be aware of students’ learning styles so as to meet students’ individual learning preferences and optimize knowledge and understanding. The aim of this study was to compare the effects of the conceptual map and the traditional lecture methods on students’ learning based on the VARK learning styles model. In this randomized controlled trial, 160 students from nursing, nurse anesthetics, and midwifery disciplines with four different learning styles of visual, auditory, reading/writing, and kinesthetic were selected using the convenience sampling method. Participants were randomly assigned to the intervention (conceptual map method) or control (traditional lecture method) groups. A medical-surgical nursing course was taught to the students in both groups over 6 weeks. Data collection tools consisted of the VARK questionnaire and pre- and postassessments. Data were analyzed using descriptive and inferential statistics via the SPSS software. Teaching using the conceptual map method had different effects on the students’ learning outcomes based on their learning styles. The conceptual map method had a statistically significant impact on the students’ learning in the intervention group compared with the control group in the students with a visual learning style (*p* = .036). No statistically significant differences were reported between the groups in other three learning styles. Nurse instructors should assess students’ learning styles based on the VARK model before the application of a particular teaching method to improve the quality of nursing education and facilitate deeper learning.

A significant gap is reported between current nursing practice and education received by nursing students in academic settings (Shinnick et al., 2011). Newly graduated nurses may have poor skills and knowledge that can pose a threat to patient safety (Duchscher, 2008). This can challenge nurse educators to provide meaningful and effective learning opportunities for students (Kaddoura, 2010). Storing information in a relational manner can be termed as meaningful learning. When a fact is recalled, associated facts are also recalled easily and immediately (Hao et al., 2013). The problem-solving approach, motivational factors, and teaching methods play important roles in meaningful learning and enhancing the quality of nurses’ performance in clinical practice (Chaghari et al., 2017). One of the most important teaching methods for facilitating meaningful learning among students is conceptual mapping (Rosen & Tager, 2018). It is a modern teaching tool and has been developed based on Ausubel’s Learning Assimilation Theory. According to this theory, meaningful learning happens if new information can be linked to preexisting knowledge using new learning materials. The use of concept maps allows new ideas to be incorporated into previous experience or knowledge (Adema-Hannes & Parzen, 2005; Ausubel, 1962). Conceptual mapping has been depicted as a bidimensional diagram consisting of concepts or knots, which are united by lines indicating relationships between them and allowing learners to arrange their knowledge through a series of graphical maps (Mih & Mih, 2011).

Teaching different concepts using conceptual maps in nursing coursework can replace other teaching methods and foster deep learning and content saturation (Vacek, 2009). Numerous studies in medical education have used conceptual maps to teach theoretical courses (Baskaran et al., 2015; Daley et al., 2016; Harrison & Gibbons, 2013). For example, Adlaon (2012), using a comparative study of 3-week teaching using conceptual maps and traditional teaching methods, studied students’ performance in answering multiple-choice questions. They reported that conceptual maps were better than traditional teaching methods. In a randomized comparison between objective-based lectures and outcome-based concept mapping for teaching neurological care to nursing students by Hsu et al. (2016), it was found that an outcome-based approach using conceptual mapping principles was more effective than objective-based lectures.

## The VARK Learning Styles Model

To better understand the effect of conceptual maps on learning, there is a need to explore the relationship between learning styles and affinity for concept mapping among students (Sciarra, 2016). Learning styles have been defined as individual learning techniques that act within the environment to process, interpret, and obtain information, experiences, or desirable skills (Othman & Amiruddin, 2010). The VARK model is one of the simplest and most convenient inventories for assessing learning styles among students (James et al., 2011). It takes into account the preferred sense of students in the process of learning (Klement, 2014). In the VARK inventory, (V) means visual: learners with preferences for graphical ways of representing information; (A) means auditory: learners with preferences for hearing information and verbal instructions; (R) means reading/writing: learners with preferences for information printed as words or taking notes during lectures; and (K) means kinesthetic: learners with preferences related to the use of experience and practical process (Fleming, 2009).

Given the different sets of characteristics of learning styles, instructors need to be aware of preferred learning styles in students for aligning teaching styles and meeting students’ individual learning preferences (Bhattacharyya & Sarip, 2014). Hsieh et al. (2011) found that those students whose learning styles were matched with the corresponding teaching style had significantly greater learning improvements than those in the mismatched groups. Hinck et al. (2006) highlighted that conceptual maps served to improve students’ abilities to see patterns and relationships for planning nursing care. It may also be helpful to students with a visual learning style.

While the significance of the use of the conceptual map over traditional methods have been reported in previous studies (Bala et al., 2016; Ghanbari et al., 2011; Pishgooie et al., 2019), few studies were found to investigate the effect of conceptual maps compared with traditional teaching methods on students’ learning in terms of learning styles. Therefore, this study aimed to compare the effects of conceptual maps against the traditional lecture method on students’ learning based on the VARK learning styles model.

## Methods

### Design

This randomized controlled study was conducted on four interventions and four control groups in a nursing and midwifery school in an urban area in the east of Iran between May and June 2018 during the second academic semester.

### Participants and Setting

Samples were chosen using a convenience method according to the following inclusion criteria: those students in nursing, nurse anesthetics, and midwifery disciplines who were enrolled on a medical-surgical nursing course with a focus on hepatic disorders (students are required to successfully complete the course to fulfill the requirements for a Bachelor degree in nursing, midwifery, and nurse anesthetics); have no prior knowledge of the conceptual map method; and willingness to take part in this study. Those students who had passed the course or were absent for more than one session of the intervention were excluded from the study.

**Figure 1.**
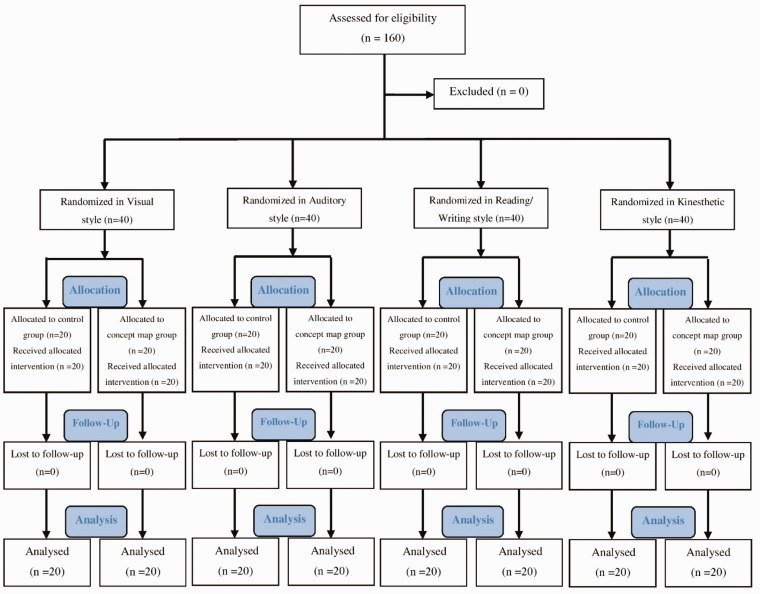
The Process of the Study.

The sample size for the present study was based on the findings of a similar study (Boström & Hallin, 2013). We calculated that a sample size of 160 students (40 students in each category of 4 different learning styles including visual, auditory, reading/writing, and kinesthetic) would enable us to detect statistically significant differences between study groups given a power = 0.80, α = .05, and an attrition rate of 10%. Accordingly, 160 students studying the medical-surgical nursing course with a focus on hepatic disorders were recruited and requested to determine their learning styles using the online VARK questionnaire: http://www.vark-learn.com/english/page.asp?p=questionnaire. Next, based on their learning styles, the participants were randomly assigned to either of four intervention groups (*n* = 20 in each group) where they were taught using the conceptual map method or four control groups (*n* = 20 in each group) who were taught using the traditional lecture method. Participants were randomized to each group by drawing a group-assignment card from a blinded box (Polit & Beck, 2010). The process of the study is shown in Figure 1.

### Data Collection

Data were collected using the VARK questionnaire (Version 7.8) and pre- and postassessments. The VARK questionnaire has been developed by Fleming, Lincoln University, New Zealand in 1998 (Fleming, 2009) and consists of 16 multiple-choice questions in the following domains of learning styles: “visual,” “auditory,” “reading/writing,” and “kinesthetic.” Each of the student’s predominant learning style or, in some cases, learning styles was indicted by the highest score. For the purpose of this study, the VARK English version was translated into Farsi, and its validity was assessed by a team of experts in the field of nursing education. In addition, reliability of this questionnaire was assessed using the Cronbach’s alpha coefficient, which was reported as .84.

Demographic data about the students’ age, type of learning style, and student’s discipline were collected. Pre- and postassessment questionnaires were developed by the researchers based on the approved curriculum and lesson plan at the faculty, which consisted of 20 questions in the domain of knowledge and remembering. These are believed to be important thinking behaviors in Bloom’s educational taxonomy (Gogus 2012). The questionnaire included items relating to recalling facts and examining the knowledge of basic concepts such as defining, listing, selecting, and memorizing. It also included 20 questions regarding the domains of understanding, applying, analyzing, evaluating, and creating as articulated in Bloom’s taxonomy and which related to meaningful learning (Krathwohl & Anderson, 2009). This part included multiple-choice questions consisting of six to seven questions for each teaching session. A total of 40 questions were designed for 6 teaching sessions, and a score of 0.5 was awarded to each question. Therefore, the minimum and maximum scores for pre- and postassessments were 0 and 20, respectively. Face and content validity of this questionnaire was assessed by eight experts from the field of medical-surgical nursing and nursing education. Its reliability was assessed using the Kuder–Richardson method (K-R21), with coefficients reported .79 in knowledge and .82 in learning domains.

### The Intervention

At the beginning of the first session, a preassessment of the students was carried out by the second researcher (V. P.). Next, during each of the six teaching sessions, all of the groups were taught a medical-surgical nursing course with a focus on hepatic disorders by one nurse instructor, who was well trained in the pedagogical approach of conceptual maps. The duration of each session was 80 minutes in the same specified classroom for 4 consecutive days per week. Teaching sessions for each teaching method was held on 2 consecutive days to avoid overcrowding of classrooms by the students and for improving learning productivity. Also, it helped distribute the effects of all the features of teaching on the quality of education such as teaching atmosphere, teaching hours, and instructors’ skills equally between the groups.

**Figure 2.**
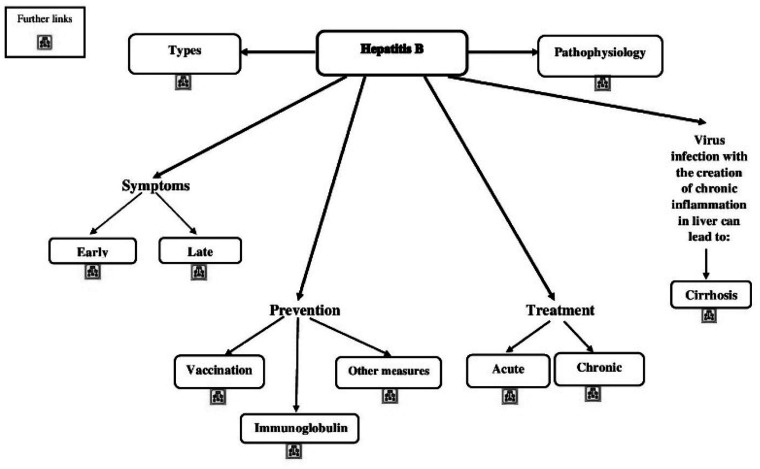
A Sample of the Conceptual Map.

In the control group, the traditional lecture method was used as the teaching method. Teaching in the intervention groups by using the conceptual map method was conducted in three phases, and a sample of this process was presented in Figure 2. Teaching of each topic on hepatic disorders consisting of various liver diseases included all three phases regardless of the number of sessions could take a different amount of time.

- *Pretraining phase*: the conceptual map method as a means of presenting the outlines of course content for creating a subjective educational background for the students.
- *Midtraining phase*: the use of conceptual maps as a means of providing course content through a full description of outlines.
- *Posttraining phase*: the use of conceptual maps as a means of summarizing and integrating course contents for the students.

After the completion of the six teaching sessions, the postassessment of students in all the groups was performed at the same time to compare the effects of the teaching methods related to all four learning styles.

### Ethical Considerations

Ethical permission was obtained from the Ethics Committee affiliated with the University in which the second author worked under the code of IR.IRSHUMS.REC.1395.8. Prior to the research, the aim of the study was explained to all students. They were assured that their information would be treated as confidential, and the findings would be used only for research purposes. Also, students were informed that participation was voluntary, and they could withdraw from the study at any phase of the research process without any effect on their educational career. Last, they were asked to sign the informed written consent form. The permission to use the VARK questionnaire (Copyright Version 7.8 (2014) held by VARK Learn Limited, Christchurch, New Zealand) was obtained from the copyright holders before the study.

### Data Analysis

Data were analyzed using descriptive and inferential statistics via the SPSS software, version 22.0 for Windows (SPSS Inc., Chicago, IL, USA). Chi-square and the independent *t* test were used for the assessment of the relationship in demographic data between the groups. The homogeneity of the students in the intervention and control groups was examined using the Skewness indexes and Kolmogorov–Smirnov test. With regard to the effectiveness of the teaching methods between the groups, differences in the pre- and postassessment scores were determined and compared using the *t* test method. The significance level was set as *p* < .05.

## Results

The demographic characteristics of the students are shown in Table 1. The majority of the students (58.7%) were female. In addition, 50% of all the students were studying nursing, 27.5% were studying midwifery, and 22.5% were studying nurse anesthetics. The age of the students ranged from 19 to 21 years with a mean of 19.73 years (*SD* = 0.68). No statistically significant differences between the intervention and control groups in terms of age, gender, and discipline were reported. No statistically significant differences were observed between the groups in terms of the demographic characteristics and preassessment mean scores. Therefore, the homogeneity of the students before the intervention was confirmed (Table 1).

**Table 1.** Demographic Characteristics of the Students in the Groups.

Variable/groups	Visual		Auditory		Reading/writing		Kinesthetic		Total
	Intervention	Control	Intervention	Control	Intervention	Control	Intervention	Control	
	*N* (%)	*N* (%)	*N* (%)	*N* (%)	*N* (%)	*N* (%)	*N* (%)	*N* (%)	*N* (%)
Gender									
Male	5 (38.5)	8 (61.5)	12 (66.7)	6 (33.3)	9 (50)	9 (50)	10 (58.8)	7 (41.2)	66 (41.3)
Female	15 (55.6)	12 (44.4)	8 (36.6)	14 (63.4)	11 (50)	11 (50)	10 (43.5)	13 (56.5)	94 (58.7)
Statistics	*p* = .311 χ^2^ = 1.026		*p* = .057 χ^2^ = 3.363		*p* = .999 χ^2^ = 0.0001		*p* = .337 χ^2^ = 0.921		
Student’s discipline									
Nursing	12 (44.4)	15 (55.6)	14 (48.3)	15 (51.7)	13 (41.9)	18 (58.1)	13 (41.9)	18 (58.1)	116 (72.5)
Nurse anesthetics/ midwifery	8 (61.5)	5 (38.5)	6 (54.5)	5 (45.5)	7 (77.8)	2 (22.2)	7 (77.8)	2 (22.2)	44 (27.5)
Statistics	*p* = .311 χ^2^ = 1.026		*p* = .723 χ^2^ = 0.125		*p* = .064 *F* = 3.584		*p* = .240 *F* = 1.129		
Age									
*M* ± *SD*	19.9 ± 0.6	19.7 ± 0.65	19.6 ± 0.67	19.5 ± 0.68	19.8 ± 0.67	19.8 ± 0.76	19.7 ± 0.65	19.7 ± 0.78	19.73 ± 0.68
Statistics	*Z* = –0.999 *p* = .383		*Z* = –0.816 *p* = .478		*Z* = –0.294 *p* = .799		*Z* = –0.088 *p* = .947		

**Figure 3.**
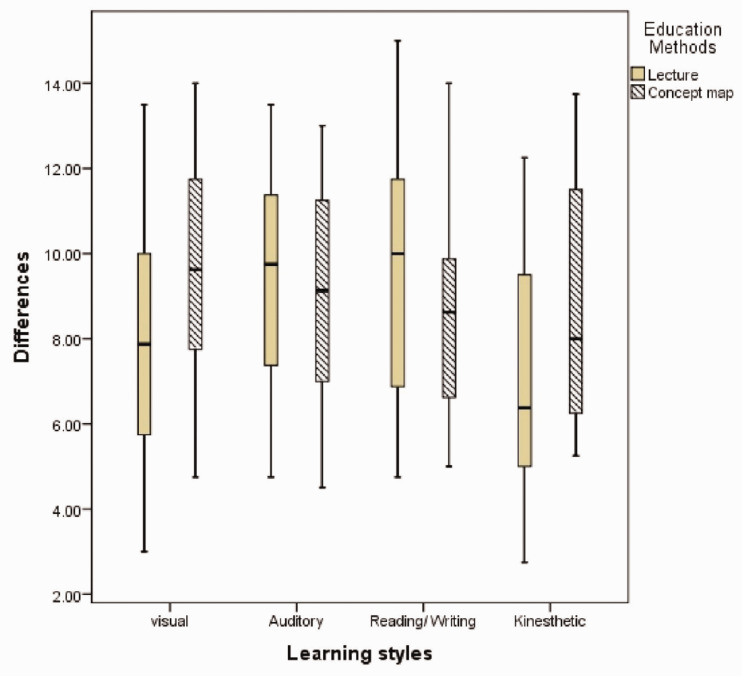
Differences Between the Means and Medians of Learning Styles.

A schematic model of differences between the means and medians of different learning styles in two teaching methods is shown in Figure 3. In each box plot, sides presented the highest and lowest learning scores, and the middle section indicates median scores. According to Figure 3 and Table 2, teaching by the conceptual map method had statistically significant differences with the control groups in the visual learning style (*p* = .036). However, no statistically significant differences were reported among the groups participating in the conceptual map method compared with the traditional lecture method in the following three learning styles including reading/writing (*p* = .414), auditory (*p* = .249), and kinesthetic (*p* = .078).

**Table 2.** Mean and Standard Deviation of the Pre- and Postassessment Scores of Learning Styles.

Learning styles	Control and intervention groups	*M* ± *SD*			*t* test	*df*	*p* value
		Preassessment	Postassessment	Difference			
Visual	Lecture	8.85 ± 2.59	16.83 ± 1.58	7.98 ± 2.77	–2.17	38	.036
	Conceptual map	9.01 ± 2.19	18.84 ± 1.18	9.83 ± 2.61			
Auditory	Lecture	9.03 ± 2.27	18.34 ± 1.38	9.31 ± 2.57	0.804	38	.249
	Conceptual map	8.94 ± 2.14	18.05 ± 1.39	9.11 ± 2.50			
Reading/writing	Lecture	8.58 ± 2.19	18.10 ± 1.40	9.26 ± 2.87	0.826	38	.414
	Conceptual map	8.70 ± 2.31	17.50 ± 1.52	8.80 ± 2.68			
Kinesthetic	Lecture	9.09 ± 2.06	16.18 ± 1.36	7.09 ± 2.71	–1.809	38	.078
	Conceptual map	8.99 ± 2.46	17.68 ± 1.33	8.69 ± 2.88			

## Discussion

This study aimed to compare the effects of the conceptual map and the traditional lecture methods on students’ learning based on the VARK learning styles model. Our results showed that teaching through the conceptual map method had a statistically significant impact on students’ learning compared with traditional lecture in the visual learning style.

The conceptual map method is a graphical teaching and learning method that helps with understanding complex information and facilitate creativity and reflective critical thinking. It is a valuable method to educate nurses in clinical settings (Chabeli, 2010). It is believed that some students may assimilate knowledge better when received from predominantly visual, auditory, or through a particular sense (Franzoni & Assar, 2009). More specifically, different learning styles need different teaching strategies for effective learning (Schmeck, 2013). Accommodating teaching methods in students’ learning styles improves students’ overall learning outcomes (Gilakjani, 2011).

However, our findings on the significant and enhancing effect of conceptual maps on learning were consistent with those of previous studies (Harrison & Gibbons, 2013; Jaafarpour et al., 2016; Van Bon-Martens et al., 2014). The findings can be discussed from two different but complementary aspects. First, teaching based on the conceptual map method had more effect on students’ learning than the traditional lecture method in the visual style. The visual format of conceptual maps may have been able to evoke complex components and abstract concepts that are usually difficult to incorporate into the formation of a mental model (Bobek & Tversky, 2016). This finding supports the assertions of Hinck et al. (2006) indicating that conceptual mapping is a graphical technique, and students with visual learning styles prefer this method more than students with strong auditory or kinetic learning styles. However, this finding is open to debate and scrutiny as other students showed different levels of compatibility between the conceptual map method and learning style, which might affect their learning.

According to the assessment of the average scores of the students, the conceptual map method among students with a predominantly visual style of learning led to higher scores in comparison to other learning styles in all groups. Nevertheless, when students with the visual learning style were taught by the traditional lecture method, they achieved lower mean scores than those students with other learning styles such as auditory and reading/writing. Interestingly, this was also true for those students with the auditory learning style. The highest average score for those students who were exposed to the lecture method, despite a nonsignificant difference, was related to the auditory style, as well as the reading/writing style. One possible reason for these results is the compatibility or the incompatibility between the teaching methods and the learning styles in the students. According to Fleming (2014), students with the visual learning style receive sensory information by ideally looking at a graphical format. In comparison, auditory learners deal best with the highly structured teaching process, including traditional and didactic lectures. In addition, those students who showed the strongest learning capabilities and were categorized as having a reading/writing learning style might have learnt the best through traditional methods such as textbook reading and lecture note-taking. The lowest average score was achieved by the kinesthetic students in the lecture group, probably due to their preferences for learning through performance activities. This finding is compatible with the Dobson’s (2009) study that the scores achieved by students with the kinesthetic style in the lecture group were lower than those in other three groups.

### Strengths and Limitations of the Research

The strength of this research is that it highlights a previously underexplored assessment of the effects of conceptual mapping on learning according to students’ different learning styles. Also, the randomization of groups to intervention and control groups to control the effects of confounding effects was another strength of this study.

A limitation of the current study was the lack of availability of similar national or/and international studies in the context of the present research. This hindered the extensive comparison of our findings with those of other studies. A small amount of evidence (Almigbal, 2015; Amira & Jelas, 2010) showed that gender was a confounding variable that might affect student learning styles. However, this line of inquiry was not possible in our study due to lack of access to equal number of males and females. Furthermore, owing to the small sample size of nursing students in each learning style, students from midwifery and nurse anesthetist disciplines were recruited. Therefore, more studies should be conducted in health sciences schools with a larger sample size comprising students from different health care disciplines. Moreover, an equal representation of gender would facilitate the generalization of findings.

### Implications for Practice

Along with most of the available references that support the use of conceptual maps as a useful teaching strategy to promote meaningful learning, this study provides a new standpoint about this teaching method. According to our result, the conceptual map is useful for students with a visual learning style but not necessarily so for all learning styles. Therefore, nurse educators are advised to apply the conceptual map in combination with the traditional lecture method to accommodate those students who are visual learners and also those with other learning styles. However, further studies are needed to assess which teaching method is the best for each individual learning style to promote student’s learning.

### Conclusion

The results of the present study indicated that teaching by the conceptual map method affected the student learning outcomes differently in terms of the visual learning style based on the VARK model. Our study findings provide evidence regarding the effectiveness of the use of the conceptual map as a teaching method in nursing and midwifery schools for creating a comprehensive and meaningful image of the nursing care process for visual learners. It is also important for nurse instructors to assess students’ learning styles before the application of a particular teaching method with the aim of improving learning outcomes and facilitating deep learning.
